# Case Report: A long-term survival case of primary malignant melanoma of the lung with meningeal metastasis

**DOI:** 10.3389/fonc.2025.1491350

**Published:** 2025-08-08

**Authors:** Ying Wu, Yanchun Wei, Xiang Meng, Yufu Zhou

**Affiliations:** Department of Radiation Oncology, The First Affiliated Hospital of Bengbu Medical University, Bengbu, Anhui, China

**Keywords:** immunotherapy, radiotherapy, melanoma, lung neoplasms, brain neoplasms

## Abstract

**Background:**

Malignant melanoma originating in the lungs is relatively rare, accounting for only 0.01% of lung tumors, and has a poor prognosis and is prone to distant metastasis. Notably, melanoma is the third most common source of intracranial metastases in adults, after lung and breast cancer. Primary malignant melanoma of the lung with meningeal metastasis is more rare and has a worse prognosis. There is no effective and unified treatment plan in clinical practice, and the main treatment is comprehensive treatment, which mainly includes surgery, radiation therapy, immunotherapy and targeted therapy.

**Case presentation:**

The patient was a 52-year-old male with no history of smoking. PET-CT showed a mass in the main bronchus of the right lung, consistent with the metabolic changes of malignant melanoma. Combined with pathology, we finally identified the patient as a primary pulmonary malignant melanoma. About two years later, the patient developed meningeal metastases. The patients was mainly treated with radiation therapy combined with immunotherapy, and the overall survival was about 30 months.

**Conclusions:**

The diagnosis of pulmonary primary malignant melanoma requires a combination of many factors and is easy to be misdiagnosed. At present, there is no specific effective treatment plan, and such patients have a poor prognosis, are prone to distant metastasis, and their survival will be greatly shortened. The survival period of the patient in this case is longer than the average survival period of such patients, and the possibility of benefiting from immunotherapy is higher. Radiotherapy also reduces the discomfort symptoms of the patient to a certain extent, prolongs the survival of the patient, and provides certain ideas for clinical treatment.

## 1 Introduction

Melanoma is a malignant transformation of melanocytes that produce pigment (1). According to GLOBOCAN statistics, there were approximately 324635 new cases of skin melanoma reported globally in 2020, with 57043 deaths from this disease (2). If melanoma is in the basal layer of the skin epidermis, it is called cutaneous melanoma, which is more common. However, due to the presence of melanocytes in the eyes, ears, gastrointestinal tract, genitalia, urinary system, and meninges, mucosal melanoma, or other types of melanomas (such as the eye) may also occur (3). Non skin melanoma presents as a lump in the primary site, which is prone to blood transmission and has a poor prognosis. Malignant melanoma originating from the lungs is rare, accounting for only 0.01% of lung tumors (4). It is worth noting that melanoma is the third most common source of adult intracranial metastasis, second only to lung cancer and breast cancer (5).

## 2 Case report

**Figure 1:**
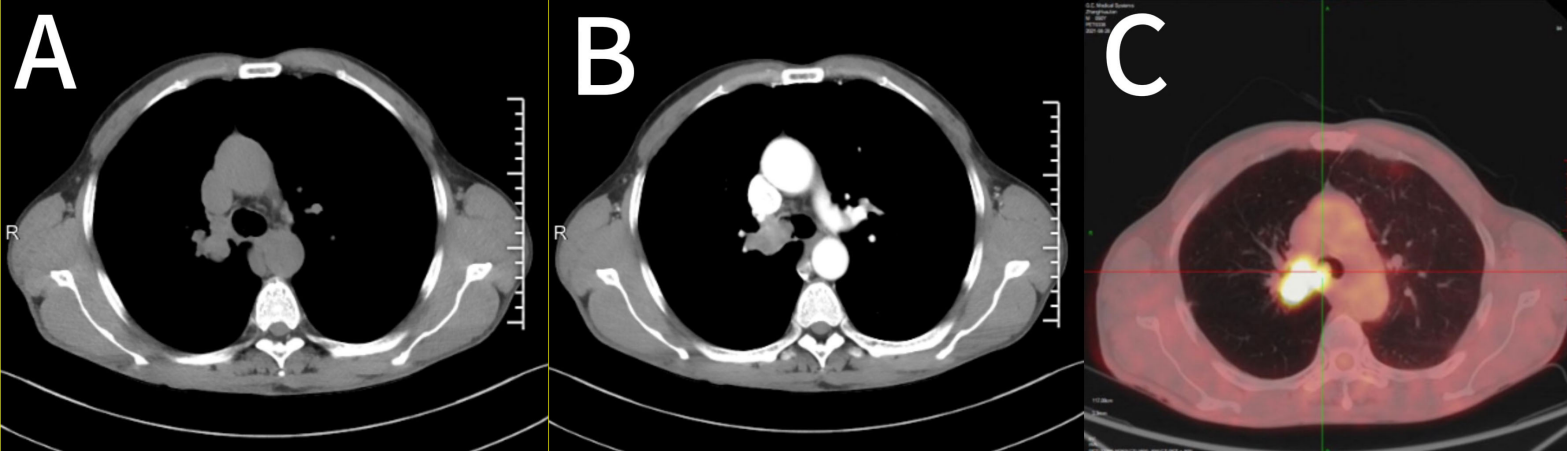
Imaging features of the case. **(A, B)** Chest CT showed a right main bronchial mass, approximately 27×32 mm. **(C)** PET-CT showed a radioactive catheter uptake mass in the lumen of the main bronchial tube of the right lung. CT, computed tomography; PET, positron emission tomography.

**Figure 2:**
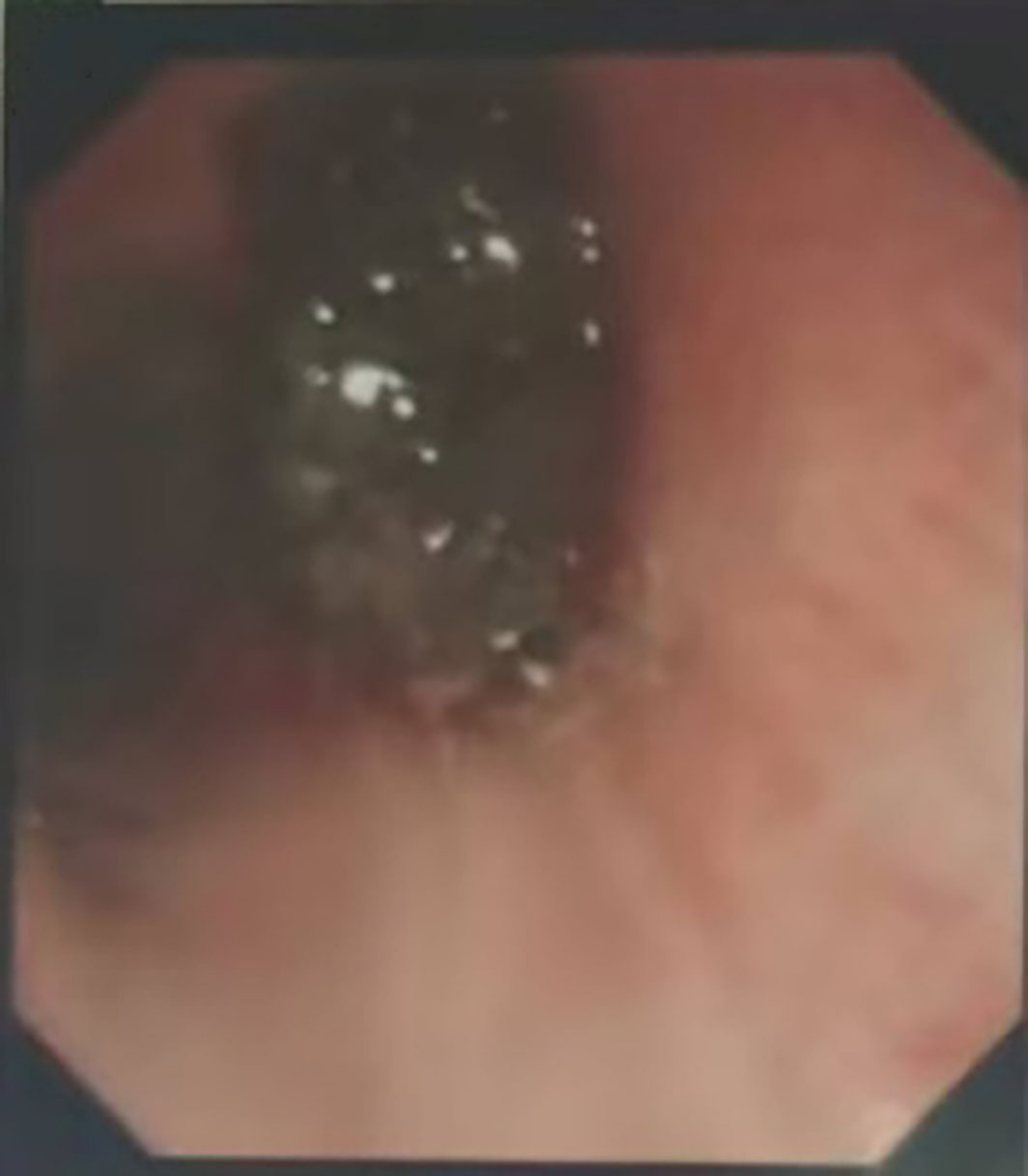
Bronchoscopic findings of the patient: Gray and new brown organisms can be seen in the main bronchus, completely blocking the lumen.

**Figure 3:**
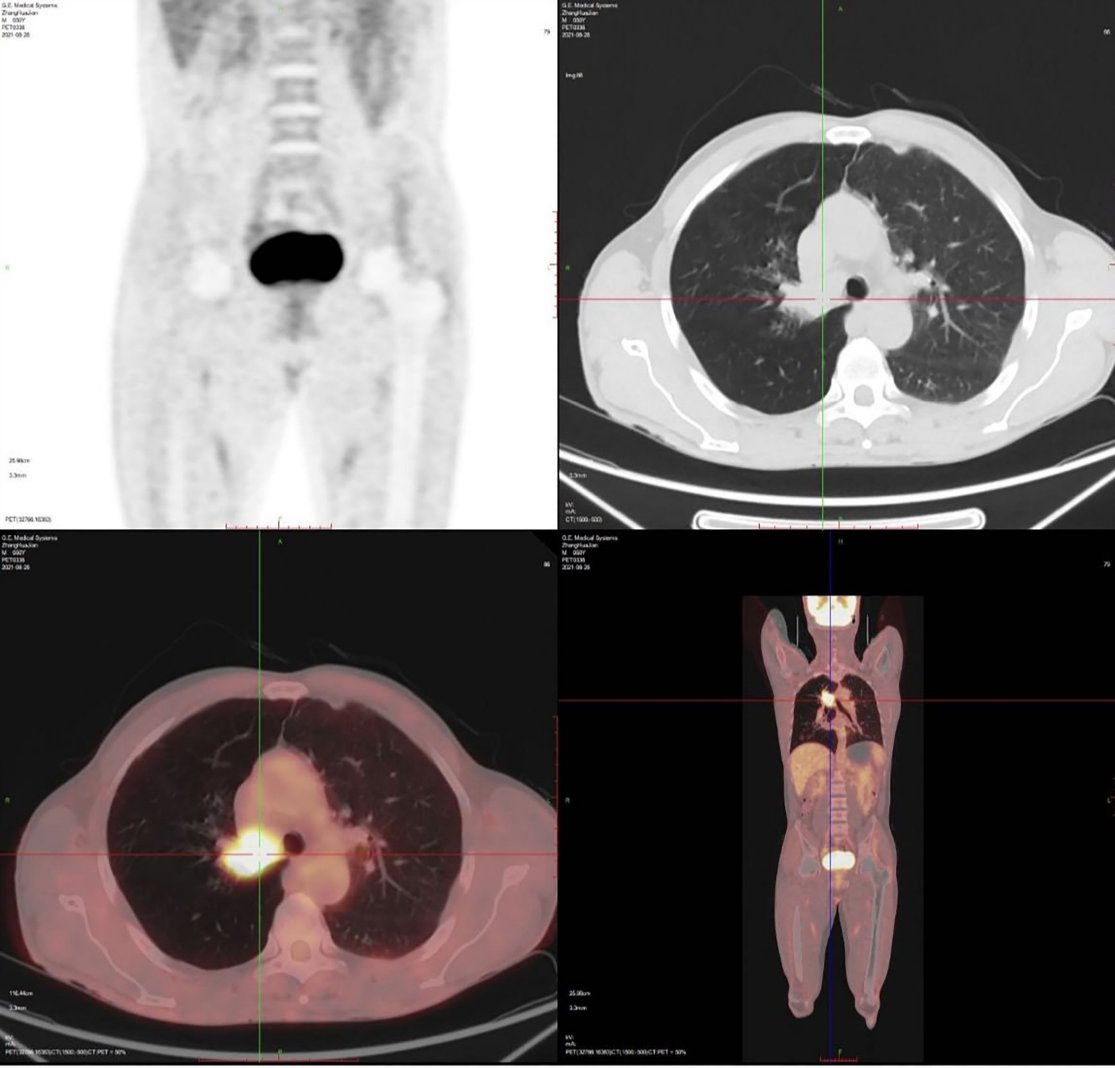
PET-CT showed there were only isolated lung lesions in the whole body.

**Figure 4:**
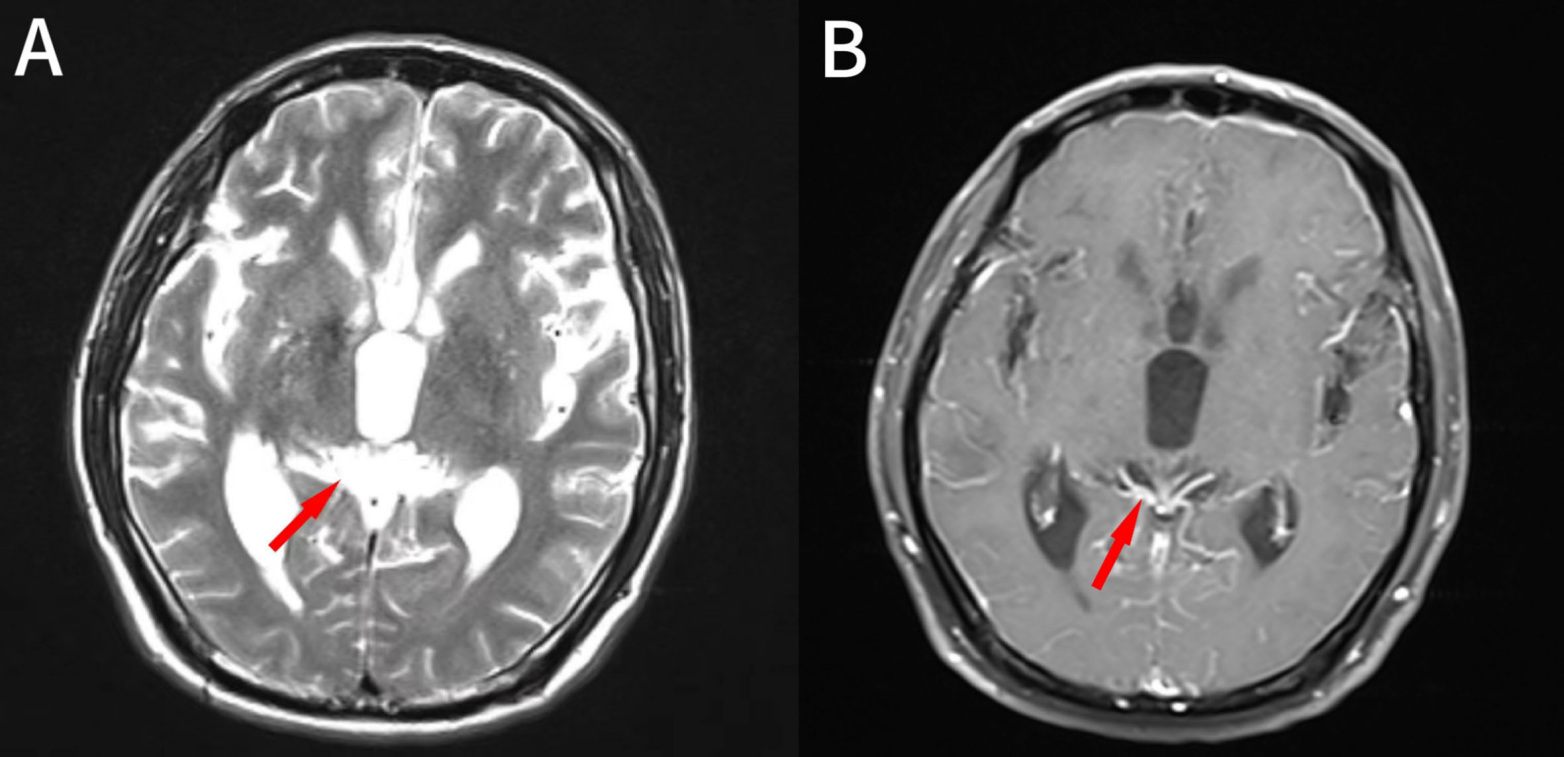
**(A, B)** The patient’s cranial MRI shows meningeal enhancement.

1.1 Case information: The patient is a 52-year-old male who first sought medical attention in August 2021 due to chest tightness, shortness of breath, and hemoptysis. Chest CT showed an increased mass density shadow in the right main bronchus, about 27×32mm, and uneven enhancement could be seen after enhancement, which was considered malignant tumor (**Figures 1A, B**). Local bronchoscopy examination in August 2021: A grayish new brown organism is seen in the right main bronchus, completely obstructing the lumen. Biopsy pathology: (right main bronchus) tissue fragmentation, with heterogeneous cell nests visible under microscope (**Figure 2**). Immunohistochemistry: HMB45 (+), S-100 (+), Melan9A (+), CK (-), Ki67 (hot spot about 60%), LCA (-), CK5/6 (-), P40 (-), P63 (-), NapsinA (-), TTF-1 (-), consistent with the immunohistochemical results of primary melanoma. Subsequently, PET-CT examination was performed on the patient, which showed that a radioactive uptake mass was visible in the lumen of the main bronchus of the right lung, with a maximum SUV of 11.7(**Figures 1C**, **3**). The patient subsequently underwent chest lesion radiotherapy with a prescription dose of 35F * 2GY, targeting lung lesions. One month after the radiotherapy, a follow-up chest CT scan showed that the tumor was smaller than the frontal area, with a reduced length and diameter (30% of the original tumor length and diameter). The efficacy was evaluated using partial remission (PR). The radiotherapy ended on September 4th, 2021. From September 2021 to December 2022, Xindilimab 200mg 3W ivgtt immunotherapy was administered for a total of 21 cycles. The immunotherapy was accompanied by two cycles of chemotherapy with the “Dacarbazine 400mg d1–3 ivgtt” regimen. However, patients refused to continue chemotherapy due to obvious side effects of chemotherapy. In December 2022, the patient developed unnoticed symptoms of dizziness and headache. On July 13, 2023, a head MRI indicated pial metastasis (**Figures 4A, B**). A lumbar puncture was performed on July 18, 2023, and the cytological examination of cerebrospinal fluid stripping showed no cell components. Chromosome copy number analysis of cerebrospinal fluid showed abnormalities. All of them suggested the possibility of mild meningeal metastasis.

**Figure 5:**
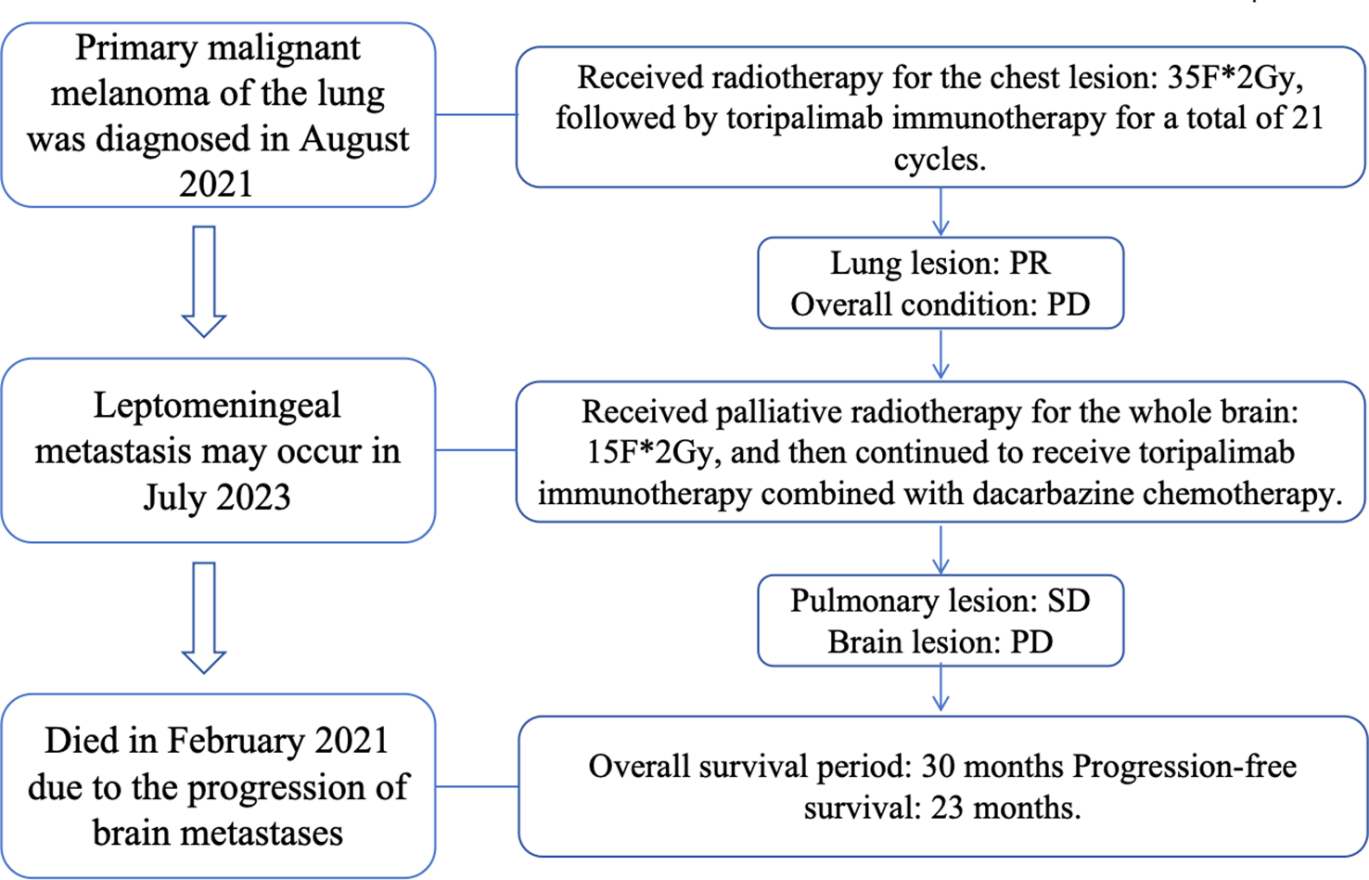
The chronological chart of the patient’s diagnosis and treatment.

1.2 Diagnosis and treatment process: The patient came to our hospital with the main complaint of “dizziness, headache for more than 1 year”. Physical examination showed black patchy nevi on both upper limbs, anterior chest, and forehead, with hair growth in the middle, and no obvious enlargement of superficial lymph nodes. Our hospital’s brain magnetic resonance imaging shows partial meningeal enhancement with dilation of the supratentorial ventricular system, considering meningeal metastasis. Based on the patient’s medical history and relevant imaging evidence, the diagnosis is primary melanoma of the right lung after radiotherapy and immunotherapy. The systematic treatment is mainly a combination of chemotherapy and immunity, and palliative radiotherapy is used for meningeal metastases. The specific target range is CTV includes the whole brain, PTV is 3mm outside the CTV, dose: 3.0Gy/F, DT: 30GY/10F; During the radiotherapy period, considering the patient’s overall tolerance, a single drug regimen of 200 mg D1 Q6W PO was administered to the patient with simvastatin capsules, followed by one cycle of chemotherapy. After one month of radiotherapy, the head MRI lesions unchanged from the prior scan, and the efficacy was evaluated using stable disease (SD). We have made a treatment time sequence chart (**Figure 5**) based on the time sequence of the patients’ diagnosis and treatment to ensure that the treatment line for the patients is clear.

## 3 Discussion

### 3.1 Clinical characteristics and pathogenesis of PMLL

The initial symptoms of PMLL are often cough, hemoptysis, difficulty breathing, chest pain (6). Usually, the sensitivity and specificity of CT, MRI, and 18F-FDG PET CT in describing melanoma depend on size and location. However, there are currently no indications for imaging in primary diagnosis (7). Black phlegm is considered an important indicator for the diagnosis of malignant melanoma. Black phlegm is caused by diffuse infiltration of melanoma cells in the lungs, as well as secondary pigment deposition by macrophages and bronchial epithelial cells (8). At present, the pathogenesis of PMML is still controversial. There are several theories that can explain its occurrence (9–11): (1) melanocytes exist throughout the body as dispersed neuroendocrine system cells; (2) Melanocytes migrate to the respiratory tract during embryonic formation and transform into malignant cancer cells in the respiratory tract; (3) Melanocytes may originate from melanocytes and produce PMML, as they share the same origin as other melanocytes located in the trachea, esophagus, and pharynx (12); (4) Melanoma cells may originate from pluripotent stem cells. More research is needed to elucidate the pathogenesis of PMML.

### 3.2 Pathological characteristics and diagnostic criteria of PMLL

The pathological manifestations of PMLL are consistent with the morphological features of malignant melanoma of the skin, mainly characterized by the presence of melanin particles in the cytoplasm and between cells. Commonly used immunohistochemical markers include HMB-45, Melan-A, and S-100 (8, 13). HMB-45 is very specific for melanocytic tumors, but its sensitivity for melanoma is lower (70%-90%) than that in S100. Melan-A has a sensitivity of 85%-97% for primary and 57%-92% for metastatic melanoma and a specificity of 95%-100%. S-100 was the first marker that proved to be useful in the diagnosis of melanoma. The sensitivity is very high (93%-100%), and S100 should be used in association with other markers (14). For the diagnosis of primary malignant melanoma of the lung, the diagnostic criteria proposed by Wilson et al. have been adopted in recent years (4): (1) solitary lung tumor; (2) Pathologically confirmed malignant melanoma; (3) No history of skin, mucosal or eye surgery or electrocautery; (4) Central lung injury; (5) At the time of diagnosis, there were no tumors in other parts.

At present, there are no clear detailed rules for the diagnosis of PMML, and it mainly relies on exclusional diagnosis. Firstly, non-metastatic malignant melanoma should be determined (excluding primary lesions such as the skin and mucosa). Secondly, it should be differentiated from other malignant tumors such as small cell lung cancer, non-small cell lung cancer, and primary pulmonary lymphoma (15). In this case, the patient did not undergo multidisciplinary joint evaluation for differential diagnosis. The diagnosis was made only by combining the imaging results such as PET-CT of the patient, as well as the relevant results of pathology and immunohistochemistry. In addition, there was a “mole” on the patient’s body surface, but no further examination was conducted. The results of biopsy or dermoscopy should include or be processed. These may lead to non-standard diagnosis and treatment. In addition, in this case, although the result of the patient’s cerebrospinal fluid cytology examination was normal, there was an abnormality in the chromosome copy number. The discovery of tumor cells in cerebrospinal fluid cytology is the gold standard for diagnosing meningeal tumors. However, the positive rate of single lumbar puncture cerebrospinal fluid cytology in the diagnosis of meningeal carcinomatosis is limited, which is not conducive to the timely diagnosis of this disease. Recent studies have found that the analysis of chromosomal copy number variations in mNGS of cerebrospinal fluid is helpful for identifying malignant tumors of the central nervous system. In this case, we further combined the imaging manifestations of the patient’s head and diagnosed it as leptomeningeal metastasis of primary malignant melanoma of the lung.

Advances in systemic treatment of local and metastatic melanoma have significantly improved the survival rate of patients. Surgery remains the main treatment method for local and regional diseases (16). Postoperative chemotherapy, radiotherapy, immunotherapy and checkpoint inhibitors, as well as targeted therapy (signal transduction and angiogenesis inhibitors, oncolytic virus therapy) are typically used to treat melanoma. Once distant metastasis occurs, the opportunity for surgery is lost. To date, the most effective treatment for metastatic melanoma is immune checkpoint inhibitors (ICI) (17). The incidence of brain metastasis in advanced melanoma is 10-40% (18). Brain metastasis remains the main complication of metastatic melanoma and causes up to half of melanoma deaths (19). However, patients with untreated brain metastases from primary malignant melanoma of the lung have a shorter survival period (20). It is still unclear how best the existing therapies can be used to prevent or treat brain metastases in patients with melanoma. Multiple studies have found that compared with patients treated with targeted therapy, patients with stage III or IV BRAF mutant melanoma treated with immunotherapy have significantly longer OS (21). Additionally, the addition of immunotherapy to radiotherapy is associated with improved OS (22). In this case, the patient mainly relied on immunotherapy and radiotherapy. Because the patient refused to undergo genetic testing, the opportunity for targeted therapy might have been lost. However, a long-term survival of nearly 30 months was still achieved here. During the follow-up process, we learned that although the brain lesions of the patient did not regress significantly, the symptoms of the central nervous system completely disappeared and the quality of life improved. And the Karnofsky Performance Status (KPS) of the patients remained good. Studies have shown that radiotherapy can increase the permeability of the blood-brain barrier, activate anti-tumor immunity by releasing tumor antigens, and change the pro-inflammatory microenvironment of tumors (23). The response to immunotherapy depends on preexisting tumor infiltrate and may be improved by radiotherapy, which is able to activate an antitumor immune response. Moreover, immunotherapy has proven to synergize with radiation-induced immune activation and to convert the immunosuppressive microenvironment of a tumor into an *in situ* vaccine (24), boosting the abscopal effect, which is defined as the clinical observation of tumor responses outside the irradiated field (25). In this study, Toripalimab combined with Dacarbazine was given priority treatment based on the economic status of the patients. Later, due to the inability to tolerate the side effects of chemotherapy, immunotherapy was used alone until the disease progressed. Before that, the chest lesions of the patients were well controlled. Treiprilizumab is the first immunosuppressant approved in China for the treatment of advanced melanoma. Studies have shown that the median overall survival time of treiprilizumab in patients with malignant melanoma who have failed systemic treatment has exceeded 22.2 months, and it is beneficial for different subtypes of melanoma (26).

## 4 Conclusions

In conclusion, primary pulmonary malignant melanoma reported in patients and literature is extremely rare. It has a high degree of malignancy in the early stage and is prone to metastasis. By sharing cases of primary malignant melanoma of the lungs, the aim is to enhance the diagnosis and differential diagnosis of this tumor, thereby achieving standardized diagnosis and treatment and improving the prognosis of patients.

## Data Availability

The original contributions presented in the study are included in the article/Supplementary Material. Further inquiries can be directed to the corresponding author.
