# Supplementary figures and images for: Case Report: A long-term survival case of primary malignant melanoma of the lung with meningeal metastasis
Source: Front Oncol. 2025 Aug 8;15:1491350. doi: 10.3389/fonc.2025.1491350 (PMC12370495; doi:10.3389/fonc.2025.1491350)

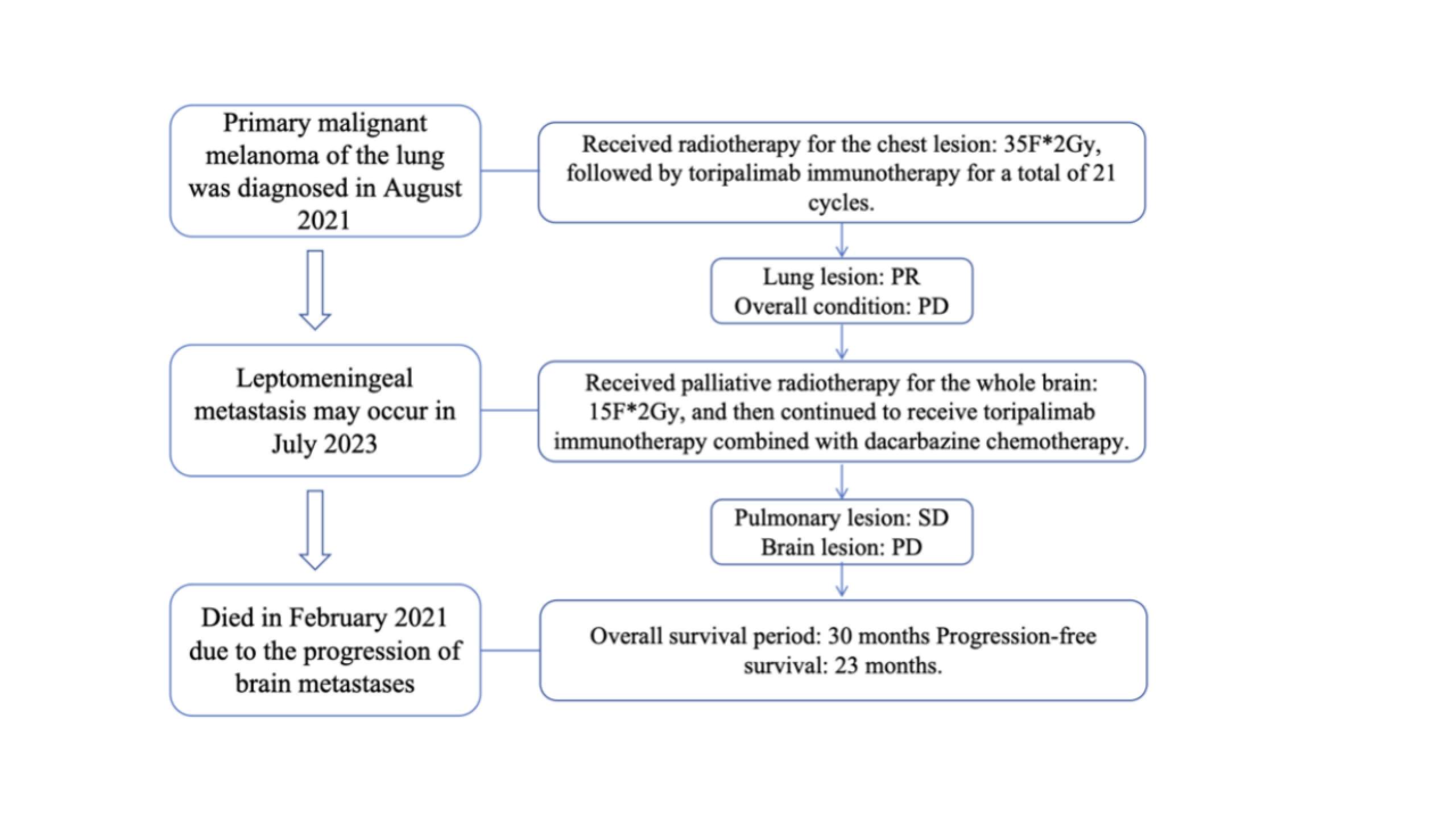

Supplement: Supplementary file 1 [file Image1.jpeg]
